# Safety and immunogenicity of inactivated SARS-CoV-2 vaccines in people living with HIV

**DOI:** 10.1080/22221751.2022.2059401

**Published:** 2022-04-18

**Authors:** Ling Ao, Ting Lu, Yu Cao, Zhiwei Chen, Yuting Wang, Zisheng Li, Xingqian Ren, Pan Xu, Mingli Peng, Min Chen, Gaoli Zhang, Dejuan Xiang, Dachuan Cai, Peng Hu, Xiaofeng Shi, Dazhi Zhang, Hong Ren

**Affiliations:** a Key Laboratory of Molecular Biology for Infectious Diseases (Ministry of Education), Institute for Viral Hepatitis, Department of Infectious Diseases, the Second Affiliated Hospital of Chongqing Medical University, Chongqing, People’s Republic of China; b The People’s Hospital of Tongliang District, Chongqing, People’s Republic of China

**Keywords:** SARS-CoV-2 vaccine, COVID-19, PLWH, safety, humoral immune response

## Abstract

It is important to know the safety and efficacy of vaccination in immunocompromised people living with HIV (PLWH), but currently, there is limited data on the inactivated SARS-CoV-2 vaccines’ safety and immune responses in PLWH. In this prospective observational study, 139 PLWH and 120 healthy controls were enrolled and monitored for 21–105 days after a two-dose vaccination. The safety, anti-receptor binding domain IgG (anti-RBD-IgG) and anti-spike-IgG responses, and RBD-specific memory B cell (MBC) responses were evaluated. The overall adverse events within seven days were reported in 12.9% (18/139) of PLWH and 13.3% (16/120) of healthy controls. No serious adverse events occurred in both groups. Overall, the seroprevalence of anti-RBD-IgG in PLWH was significantly decreased (87.1% vs. 99.2%; *p*<0.001). The geometric mean end-point titer (GMT) of anti-RBD-IgG in PLWH was also reduced, especially in patients with CD4 counts <200 cells/µL, regardless of age, gender, or HIV viral load. GMTs of anti-RBD-IgG in both PLWH and healthy controls declined gradually over time. Similar results were also observed in the anti-spike-IgG response. The frequency of RBD-specific MBCs in PLWH decreased (*p*<0.05), and then remained stable over time. Lastly, through multivariate analysis, we found the factors that predicted a less robust response to inactivated vaccines in PLWH were a low CD4 count and long time interval after vaccination. In conclusion, inactivated vaccines are well-tolerated in PLWH but with low immunogenicity. Therefore, SARS-CoV-2 vaccines and booster doses should be given priority in PLWH, especially in patients with low CD4 counts.

**Trial registration:** ClinicalTrials.gov identifier: NCT05043129..

## Introduction

The coronavirus disease 2019 (COVID-19) epidemic caused by severe acute respiratory syndrome coronavirus 2 (SARS-CoV-2) began in 2019 and has continued to rage worldwide, bringing a heavy burden to global public health [1]. People living with HIV (PLWH) may be at increased risk for severe COVID-19 due to immunosuppression and higher rates of multimorbidity; therefore, these individuals may benefit from SARS-CoV-2 vaccination [2,3]. Even though several vaccines have been recommended for PLWH [4,5], their safety and efficacy in PLWH is controversial.

Several studies indicated that PLWH have good tolerance to the ChAdOx1 nCoV-19 vaccine against SARS-CoV-2, and PLWH with well-controlled antiretroviral therapy (ART) have a similar immune response as healthy controls [6,7]. However, another study reported a suboptimal immune response in PLWH who received the Moderna vaccine [8]. PLWH with CD4 counts <200 cells/µL have diminished SARS-CoV-2 antibody production after an acute SARS-CoV-2 infection [9]. However, other studies have shown contrasting results indicating that this group can also elicit an antibody response after being vaccinated with a SARS-CoV-2 mRNA vaccine [10–12]. In addition, a case of viral activation and CD4^+^ T cell loss after receiving an inactivated COVID-19 vaccine in a treatment-naïve HIV-positive patient was reported by a recent study [13]. Therefore, the safety and immunogenicity of inactivated vaccines in PLWH are still unclear.

After vaccination, long-lasting humoral immunity is mediated by the antibody-secreting cells (ASCs) residing in the bone marrow and memory B cells (MBCs). When a secondary infection occurs, MBCs rapidly proliferate and differentiate into ASCs, protecting against severe disease or death [14,15]. However, no relevant data about the response of SARS-CoV-2-specific MBCs in PLWH after vaccination has been reported to date.

This study recruited 139 PLWH on stable ART and 120 healthy controls to evaluate the safety and SARS-CoV-2 RBD-IgG and spike-specific IgG response 21–105 days after receiving the inactivated vaccine. The response of MBCs and its four subpopulations were detected by flow cytometry.

## Materials and methods

### Participants and study design

In this prospective observational study, PLWH on stable ART from the People’s Hospital of Tongliang District, Chongqing City, and healthy controls from the health management centre of the Second Affiliated Hospital of Chongqing Medical University were recruited consecutively between 1 September 2021 and 30 November 2021. The key inclusion criteria for all individuals were: (i) 21–105 days after full-course vaccination (BBIBP-CorV [16] or Corona Vac [17]), (ii) age ≥18 years. Key exclusion criteria were: (i) history of COVID-19 infection, (ii) use of immunosuppressants within 6 months, (iii) autoimmune disease, and (iv) pregnancy.

Firstly, we conducted a cross-sectional analysis. Considering the different index dates for each vaccine recipient, we defined 21–45, 46–75, and 76–105 days as 1, 2, and 3 months, respectively, to study antibody and B cell responses over time. For the participants at 1-month, we continued follow-up until 6 months.

This study was approved by the Ethics Committee of the Second Affiliated Hospital of Chongqing Medical University and conformed with the ethical guidelines of the Declaration of Helsinki. Written informed consent was obtained from all participants. This study has been registered at www.ClinicalTrials.gov (NCT05043129) and chictr.org.cn (ChiCTR2100050922).

### Adverse events monitoring

Adverse events within 7–30 days after vaccination were self-reported by questionnaires. The classification of adverse events was based on a scale issued by the National Medical Products Administration (2019 version).

### SARS-CoV-2 antibody testing

Serum samples of all participants were taken 21–105 days after full-course vaccination. The IgG antibody against spike protein receptor-binding domain (anti-RBD-IgG) was detected by indirect ELISA using a SARS-CoV-2 RBD antibody detection kit (Sino Biological, Beijing, China). The IgG antibody against spike protein (anti-spike-IgG) was detected using a SARS-CoV-2 spike protein detection kit (Sino Biological, Beijing, China). According to the manufacturer's instruction, serum was considered seropositive for IgG binding antibodies when the OD value ≥2.1 times the mean absorbance value of negative controls at 1:50 dilutions, and the antibody titers were presented as the highest serum dilution showing a positive result. In addition, the positive result for the anti-spike-IgG was >1.495 AU/mL. The detailed procedure of each antibody is shown in the Supplementary Materials.

### Detection of SARS-CoV-2-specific B cells by flow cytometry

In order to detect SARS-CoV-2-specific B cells, biotin-labeled SARS-CoV-2 spike RBD protein (40592-V08H2-B; Sino Biological, Beijing, China) was mixed with Streptavidin BV421-11 (405225; Biolegend, California, CA, USA) at a molar ratio of 4:1 for 1 h to obtain an antigen probe. According to the manufacturer's instructions, peripheral blood mononuclear cells were isolated from whole heparinized blood by Histopaque (10771; Sigma-Aldrich, St Louis, MO, USA) density gradient centrifugation. After washing with flow cytometry staining (FACS) buffer (phosphate-buffered saline with 2% fetal bovine serum), staining was performed for 30 min at 4°C with an antigen probe (1:33.3) and the following binding antibodies at 1:50 dilution: anti-human CD3 (300430), anti-human CD19 (302212), anti-human CD21 (354918), and anti-human CD27 (356406) all purchased from Biolegend. After staining, the cells were re-washed and suspended in 200 µL FACS buffer. Samples were evaluated using a CytoFLEX cytometer (Beckman Coulter, Inc., Brea, CA, USA), and FlowJo software version 10.0.7r2 (Treestar Inc., Ashland, OR, USA) was used for data analysis.

### Statistical analysis

Statistical analysis was performed according to the type of data. Normality assumption was checked for all continuous variables. Chi-square test and Fisher's exact test were used for categorical variables. Continuous variables were compared with Mann–Whitney U-test (for unpaired data) or Wilcoxon test (for paired data) for two groups and Kruskal–Wallis test for three groups. The results of multiple comparisons were corrected with Bonferroni. Statistical analysis was performed with IBM SPSS(version 25.0). GraphPad Prism(version 9.0.0) was used for plotting. *p*<0.05 was statistically different.

## Results

### Baseline characteristics of all participants

The median ages of PLWH and healthy controls were 55 (range: 23–81 yrs) and 54 (range: 21–83 yrs) yrs, respectively. More than half of the participants were male (64.0% [89/139] in PLWH and 60% [72/120] in healthy controls). The median time for vaccine safety and immunogenicity analysis post-vaccination was 40 (range: 23–102 days) and 41 (range: 21–105 days) days for PLWH and healthy controls, respectively. There were no significant differences between the two groups after the second dose of vaccination at 1, 2, and 3 months. Of note, PLWH received combined ART for more than one year. Most PLWH (92.8%) used one non-nucleoside reverse transcriptase inhibitor in combination with two nucleoside reverse transcriptase inhibitors (NRTIs), while the remainder received a protease inhibitor-based booster regimen, with either one (4.3%) or two (2.9%) NRTIs. Of the 139 PLWH, 18 (13%) patients had a CD4 count of <200 cells/µL and 109 (78.4%) patients had an HIV viral load of <20 copies/mL. Notably, the levels of alanine aminotransferase (23.0 U/L vs. 19.0 U/L; *p*<0.001) and aspartate aminotransferase (24.4 U/L vs. 20 U/L; *p*<0.001) were significantly higher in PLWH than those in healthy controls (Table 1).

**Table 1.** Characteristics of participants.

Variable	PLWH(n=139)	HC(n=120)	*P* value
**Age(years)median(range)**	55(23-81)	54(21-83)	0.591
18-49, n (%)	46(33.1%)	47(39.2%)	0.188
≥50, n (%)	93(66.1%)	73(60.8%)	
**Gender**			
Male, n (%)	89(64.0%)	72(60.0%)	0.523
Female, n (%)	50(36.0%)	48(40.0%)	
**Days after 2nd dose** **Vaccination, median(range)**	40(23-102)	41(21-105)	0.148
1 month (21-45 days) (n, %) 2 month (46-75 days) (n, %) 3 month (76-105 days) (n, %)	96(69.0%) 13(9.4%) 30(21.6%)	70(58.3%) 23(19.2%) 27(22.5%)	0.059
**ART use^#^**			
NNRTI + two NRTIs	129(92.8%)	/	/
Boosted PI + one NRTI	6(4.3%)	/	/
Boosted PI + two NRTIs	4(2.9%)	/	/
**COVID-19 vaccine**			
BBIBP-CorV, n (%)	34(24.5%)	53(44.2%)	0.000
Corona Vac, n (%)	67(48.2%)	61(50.8%)	
BBIBP-CorV + Corona Vac, n (%)	38(27.3%)	6(5%)	
**CD4 count(cells/µL)**			
>500, n (%)	47(33.8%)	/	/
200-500, n (%)	74(53.2%)	/	/
<200, n (%)	18(13.0%)	/	/
**Plasma HIV viral load**			
>20 copies/mL, n (%)	30(21.6%)	/	/
<20 copies/mL, n (%)	109(78.4%)	/	/
**Laboratory examination**			
White blood cell count (10^^^9/L)	5.97(3.35-11.92)	5.46(3.52-9.86)	0.106
Lymphocyte count (10^^^9/L)	1.81(0.48-7.16)	1.84(0.97-3.48)	0.574
Platelet count (10^^^9/L)	211(91-713)	198(107-478)	0.385
Alanine aminotransferase (U/L)	23(8.8-116.4)	19(5-4)	0.000
Aspartate aminotransferase (U/L)	24.4(11.4-147.9)	20(9-38)	0.000

ART, antiretroviral therapy; NNRTI, non-nucleoside reverse transcriptase inhibitor; NRTI, nucleoside or nucleotide reverse transcriptase inhibitor; PI, protease inhibitors; #The majority (92.8%) of the patients were on the efavirenz regimen, while the remainder received a protease inhibitor-based booster regimen, lopinavir plus ritonavir, and one or two NRTIs, including lamivudine, zidovudine, abacavir, or tenofovir.

### Safety of inactivated SARS-CoV-2 vaccines in PLWH

As shown in Table 2, the overall incidence of adverse events within 7 days after vaccination in PLWH was 12.9% (18/139), which was similar to that of healthy controls (13.3%, [16/120]; *p* = 0.927). Injection site pain was the most common local adverse reaction, occurring in 8.6% (12/139) of PLWH and 7.5% (9/120) of healthy controls. Swelling, redness, and itch were uncommon in both PLWH and healthy controls (all <5%). Systemic adverse events in PLWH included fatigue and rash which were reported in three and two patients, respectively. Likewise, the healthy controls had scant systemic adverse reactions, including fatigue (0.8%, 1/120), drowsiness (2.5%, 3/120), cough (0.8%, 1/120), and abdominal pain (0.8%, 1/120). All adverse events were mild (grade 1 and 2) and resolved spontaneously within 7 days, according to the self-reports from all participants. After 30 days of observation, no new adverse events occurred in either group (Table 2).

**Table 2.** Adverse events of COVID-19 vaccination in participants.

Variable	PLWH(n=139)	HC (n=120)	*P* value
Overall adverse events within 7 days	18(12.9%)	16(13.3%)	0.927
Overall adverse events within 30 days	18(12.9%)	16(13.3%)	0.927
**Local adverse events**			
Pain	12(8.6%)	9(7.5%)	0.739
Swelling	1(0.7%)	4(3.3%)	0.284
Redness	1(0.7%)	1(0.8%)	1.000
Itch	1(0.7%)	1(0.8%)	1.000
Induration	/	/	1.000
**Systemic adverse events**			
Muscle pain	/	/	1.000
Pruritus	/	/	1.000
Rash	2(1.4%)	/	0.501
Fatigue	3(2.2%)	1(0.8%)	0.721
Drowsiness	/	3(2.5%)	0.098
Headache	/	/	1.000
Rhinorrhea	/	/	1.000
Laryngeal pain	/	/	1.000
Fever	/	/	1.000
Chill	/	/	1.000
Cough	/	1(0.8%)	0.463
Inappetence	/	/	1.000
Abdominal pain	/	1(0.8%)	0.463
Abdominal distension	/	/	1.000
Diarrhea	/	/	1.000
Nausea	/	/	1.000
Chest distress	/	/	1.000
Constipation	/	/	1.000
Decreased hemoglobin	/	/	1.000
Decreased platelet count	/	/	1.000
Elevated liver enzymes	/	/	1.000
Decreased albumin	/	/	1.000
**Grade 3 and 4 adverse events**	/	/	1.000

### Antibody responses to inactivated SARS-CoV-2 vaccines in PLWH

**Figure 1.**
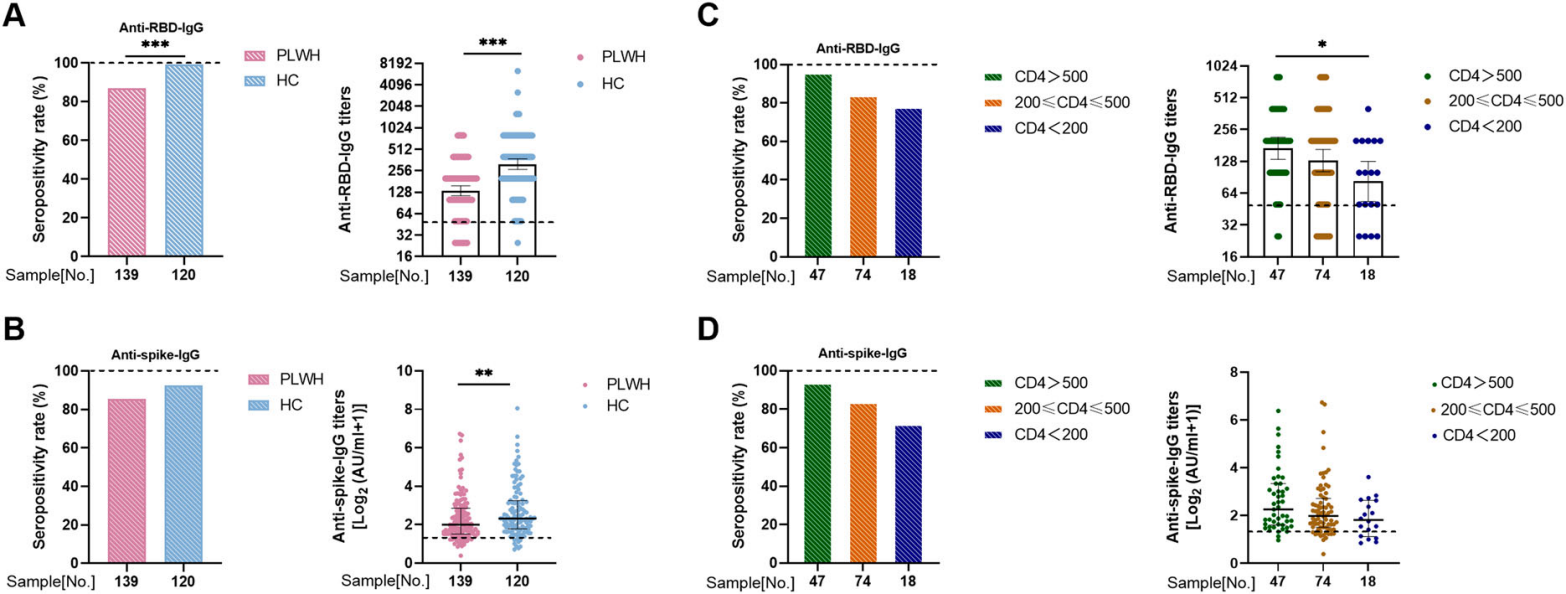
**Antibody responses to inactivated vaccines in people living with HIV (PLWH).** The seropositivity rate and titers of (**A**) anti-receptor binding domain (RBD)-IgG and (**B**) anti-spike-IgG in PLWH and healthy controls. The seropositivity rate and titers of (**C**) anti-RBD-IgG and (**D**) anti-spike-IgG in PLWH with different CD4 count levels. The horizontal dotted lines represent the limit of detection.

Blood samples from all subjects were collected at a single time point, between 21 and 105 days after full-course vaccination for cross-sectional analysis. Overall, the seroprevalence of anti-RBD-IgG in PLWH was significantly lower than that of healthy controls (87.1% vs. 99.2%; *p*<0.001). Similarly, the geometric mean end-point titers (GMTs) of anti-RBD-IgG were also significantly reduced in PLWH than in healthy controls (134.2 [95% CI: 114.0–158.0] vs. 317.5 [95% CI: 267.1–377.4]; *p*<0.001; Figure 1(A)). A similar trend was observed in seropositivity rate and titers of anti-spike-IgG. PLWH had lower anti-spike-IgG titers than healthy controls (2 log_2_ AU/mL; interquartile range [IQR: 1.51–2.85 log_2_ AU/mL] vs. 2.32 log_2_ AU/mL; IQR [1.79–3.25 log_2_ AU/mL]; *p*<0.01; Figure 1(B)). Subgroup analysis of gender and age showed that there were no differences in both seropositivity and GMTs of anti-RBD-IgG and anti-spike-IgG in PLWH (Figure S1 and S2). Further analysis of different vaccination regimens (BBIBP-CorV, Corona Vac, and BBIBP-CorV+Corona Vac) revealed a similar trend (Figure S3).

To understand whether HIV CD4 counts or viral load can influence vaccine-induced antibody responses in PLWH, we performed a subgroup analysis. Among the 139 PLWH, 18, 74, and 47 patients had CD4 counts of <200, 200–500, and >500 cells/µL, respectively. PLWH with CD4 counts <200 cells/µL elicited an antibody response to the inactivated vaccines (Figure 1(C)). However, GMTs of anti-RBD-IgG in PLWH gradually decreased as CD4 counts declined, especially in the group with <200 cells/µL (170.0 [95% CI: 133.7–216.4] vs. 82.49 [95% CI: 53.2–128.0]; *p*<0.05). A similar trend was observed in the anti-spike-IgG response (Figure 1(D)). As shown in Figure S4, there were no differences in the GMTs of anti-RBD-IgG between different HIV viral load groups (158.7 [95% CI: 112.7–223.5] vs. 128.1 [95% CI: 106.2–154.6]; *p* = 0.26). Similarly, there were no significant differences in the titers of anti-spike-IgG (1.87 log_2_ AU/mL; IQR: 1.50–2.75 log_2_ AU/mL vs. 2.03 log_2_ AU/mL; IQR 1.54–2.90 log_2_ AU/mL; *p* = 0.44; Figure S4).

Taken together, these results indicated that the antibody response to inactivated SARS-CoV-2 vaccines in PLWH was inferior, especially in PLWH with a lower CD4 count level.

### RBD-specific MBC responses to inactivated SARS-CoV-2 vaccines in PLWH

**Figure 2.**
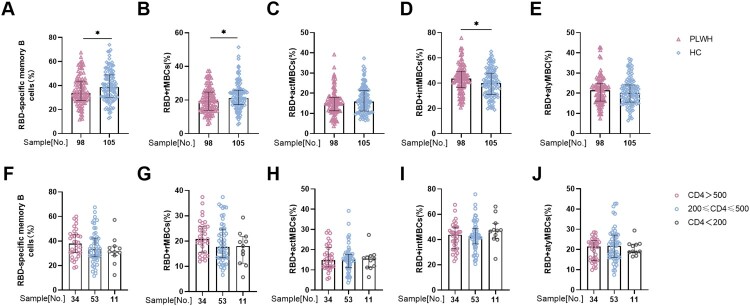
**Specific memory B cell (MBC) responses to inactivated vaccines in people living with HIV (PLWH).** The frequencies of **(A)** receptor binding domain (RBD)-specific MBCs, **(B)** resting MBCs, **(C)** activated MBCs, **(D)** intermediate MBCs, and **(E)** atypical MBCs in PLWH and healthy controls. The frequencies of **(F)** RBD-specific MBCs, **(G)** rMBCs, **(H)** actMBCs, **(I)** intMBCs, and **(J)** atyMBCs in PLWH with different CD4 count levels.

Since we proved the suboptimal antibody response in PLWH, we hypothesized that durable MBC responses were impaired in PLWH. Notably, the overall frequency of RBD-specific MBCs in PLWH was obviously lower than that in healthy controls (33.7% vs. 38.6%; *p*<0.05; Figure 2(A)). To further understand the function of MBCs, four subsets of MBCs, namely, activated MBCs (actMBCs), resting MBCs (rMBCs), intermediate MBCs (intMBCs), and atypical MBCs (atyMBCs), were analyzed in both groups. Interestingly, we found that the percentages of rMBCs (19.35% vs. 21.2%; *p*<0.05) and actMBCs (14.95 vs. 15.90; *p*>0.05) were lower in PLWH than those in healthy controls. In contrast, the percentages of intMBCs (43.5% vs. 39.9%; *p*<0.05) and atyMBCs (21.4% vs 20.1%; *p*>0.05) were higher in PLWH than in healthy controls (Figure 2(B–E)). The gating strategy and representative flow cytometric results are shown in Figure S5.

In addition, we further analyzed the response of MBCs in patients with different CD4 counts. The overall frequency of MBCs in patients with CD4 <200 cells/µL was lower than in the other two groups, but the difference was not statistically significant (Figure 2(F)). In addition, the percentages of the four MBC subsets fluctuated among the three groups (Figure 2(G–J)). In summary, RBD-specific MBC responses in PLWH were also compromised.

### Humoral immune responses to inactivated SARS-CoV-2 vaccines in PLWH over time

**Figure 3.**
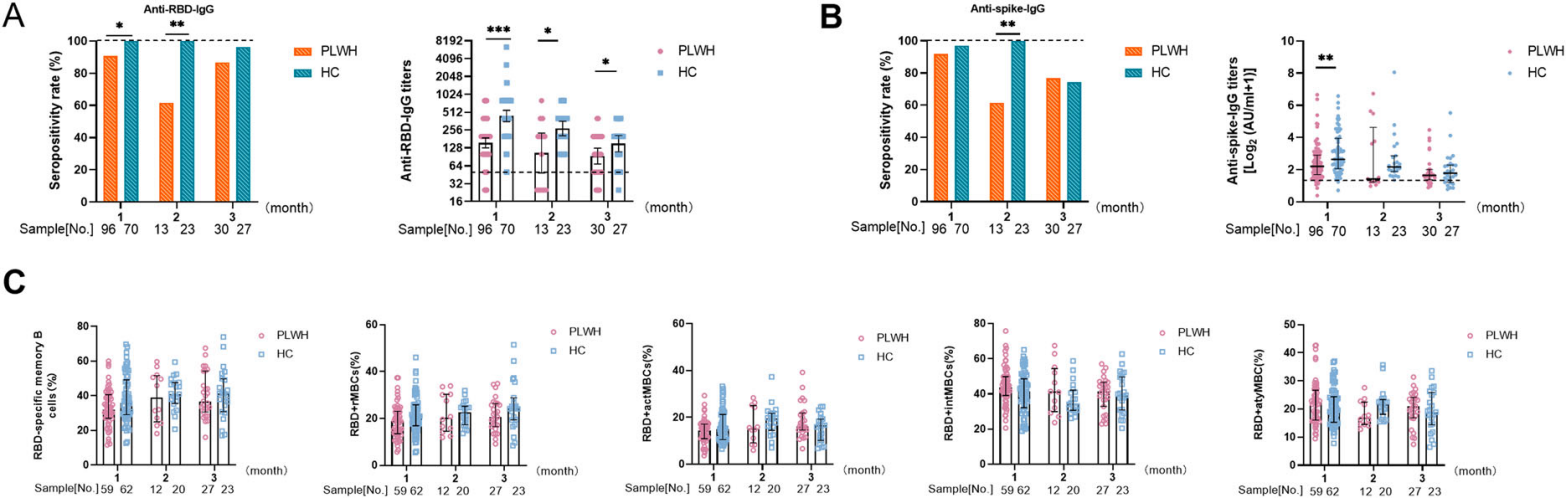
**Antibody responses and specific memory B cell (MBC) responses to inactivated vaccines over time.** The seropositivity rate and titers of anti-receptor binding domain (RBD)-IgG (**A**) and anti-spike-IgG (**B**) after 1, 2, and 3 months in people living with HIV (PLWH) and healthy controls. (**C**)The frequencies of RBD-specific MBCs, rMBCs, actMBCs, intMBCs, and atyMBCs after 1, 2, and 3 months in PLWH and healthy controls. The horizontal dotted lines represent the limit of detection.

To better understand the variation of humoral immune responses with passing time, we stratified three groups by time interval after full-course vaccination in the cross-sectional analysis. As expected, GMTs of anti-RBD-IgG gradually decreased over time in both PLWH and healthy controls. However, GMTs of anti-RBD-IgG in PLWH were sharply lower than that of healthy controls at every time point (1 month: 155.3 [95% CI: 128.3–188.1] vs. 441.6 [95% CI: 355–549.5], *p*<0.001; 2 months: 105.5 [95% CI: 48.67–228.6] vs. 278.6 [95% CI: 204.1–380.4], *p*<0.05; 3 months: 93.3 [95% CI: 68.66–126.8] vs. 150.8 [95% CI: 109.9–206.8], *p*<0.05; Figure 3(A)). Similarly, the titers of anti-spike-IgG in PLWH were lower than the titers in healthy controls at every time point (1 month: 2.21 log_2_ AU/mL, IQR [1.67–2.90 log_2_ AU/mL] vs. 2.64 log_2_ AU/mL, IQR [2.07–3.94 log_2_ AU/mL], *p*<0.01; 2 months: 1.42 log_2_ AU/mL, IQR [1.23–4.63 log_2_ AU/mL] vs. 2.17 log_2_ AU/mL, IQR [1.88–2.86 log_2_ AU/mL], *p* = 0.169; 3 months: 1.65 log_2_ AU/mL, IQR [1.38–2.01 log_2_ AU/mL] vs. 1.78 log_2_ AU/mL, IQR [1.21–2.29 log_2_ AU/mL], *p* = 0.786; Figure 3(B)) For RBD-specific MBCs, the frequency of MBCs at every time point was slightly lower in PLWH than in healthy controls, although this was not statistically significant. Furthermore, the frequency of MBCs was relatively stable in both PLWH and healthy controls over time. Similarly, the frequencies of the four MBC subsets persisted over time (Figure 3(C)).

In summary, the weakened antibody response in PLWH kept on falling over time, whereas the impaired MBC response did not change over time.

### The longitudinal dynamic changes of humoral responses to inactivated SARS-CoV-2 vaccines in PLWH

**Figure 4.**
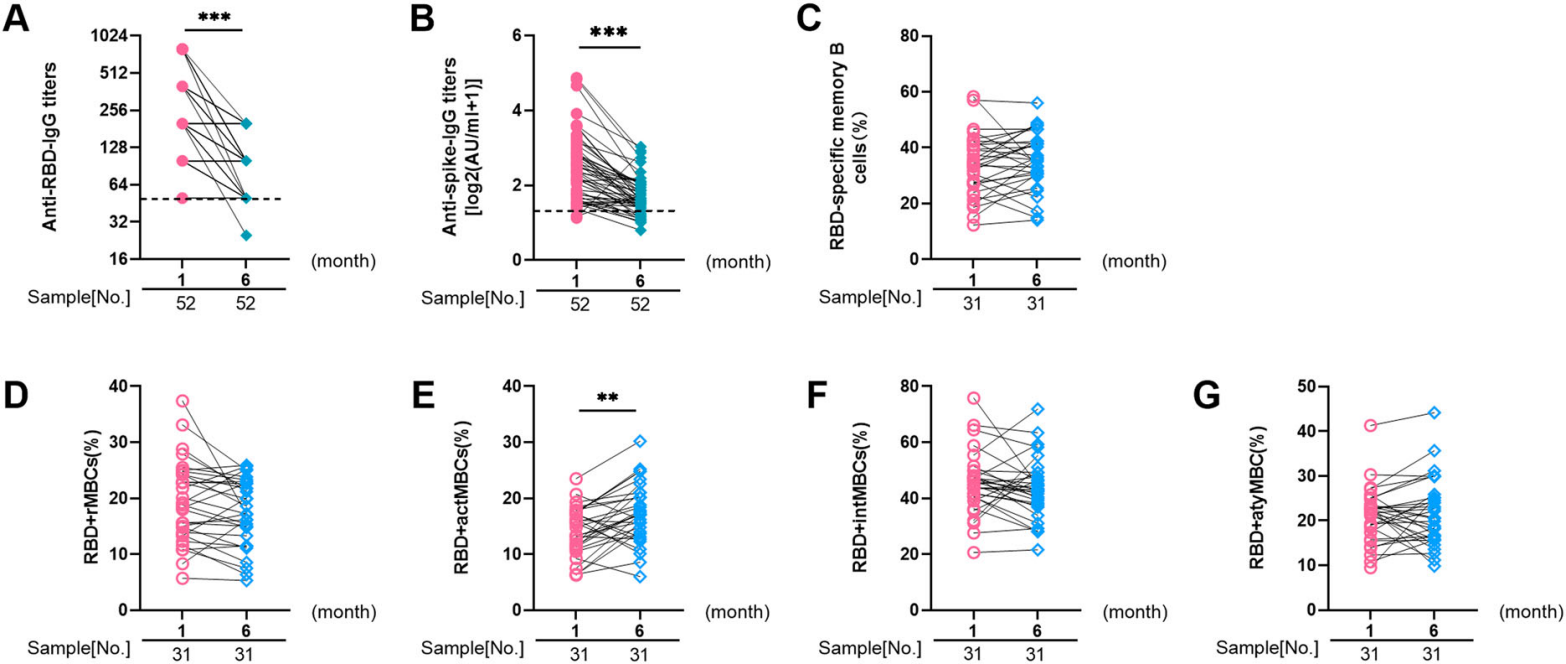
**The longitudinal dynamic changes of humoral responses to inactivated SARS-CoV-2 vaccines in people living with HIV (PLWH).** The dynamic changes of anti-receptor binding domain (RBD)-IgG (**A**) and anti-spike-IgG (**B**) titers in PLWH from month 1 to month 6 post-vaccination. The dynamic changes of frequencies of (**C**) RBD-specific MBCs, (**D**) rMBCs, (**E**) actMBCs, (**F**) intMBCs, and (**G**) atyMBCs in PLWH from month 1 to month 6 after vaccination. The horizontal dotted lines represent the limit of detection.

To further explore the dynamic changes of antibody levels in PLWH, a longitudinal analysis was conducted in PLWH. Of the 96 PLWH observed after 1 month, 52 were followed up to the 6th month. As expected, GMTs of anti-RBD-IgG showed a clear downward trend in the 6th month compared with the 1st month (219.6 [95% CI: 179.3–268.8] vs. 97.37 [95% CI: 83.99–112.9]; *p*<0.001; Figure 4(A)). Similarly, the titers of anti-spike-IgG also declined (2.38 log_2_ AU/mL, IQR [1.72–2.98 log_2_ AU/mL] vs. 1.61 log_2_ AU/mL, IQR [1.41–2.03 log_2_ AU/mL]; *p*<0.001; Figure 4(B)). The response of longitudinal RBD-specific MBC was similar to that of cross-sectional analysis. (Figure 4(C–G)).

### Factors related to poor responses to inactivated SARS-CoV-2 vaccines in PLWH

Lastly, we wanted to investigate the risk factors related to inferior response to anti-RBD-IgG in PLWH. As shown in Table 3, factors significantly related to the poor response of anti-RBD-IgG were the time interval after full-course vaccination and a low CD4 count level.

**Table 3.** Univariate and multivariate analyses for anti-RBD-IgG in PLWH.

Variable	Univariate OR (95% CI)	*P* value	Multivariate OR (95% CI)	*P* value
Age (years)	0.995 (0.970-1.020)	0.696	0.985 (0.954-1.018)	0.382
Gender (male)	0.712 (0.384-1.319)	0.280	0.963 (0.422-2.197)	0.928
Days after 2nd dose Vaccination	**0.978 (0.966-0.991)**	**0.001**	**0.969 (0.953-0.983)**	**0.000**
Plasma HIV viral load				
>20 copies/mL	1.498 (0.728-3.083)	0.272	2.104 (0.861-5.140)	0.103
CD4 count (cells/µL)				
<200	**0.284 (0.106-0.758)**	**0.012**	**0.206 (0.053-0.797)**	**0.022**
200–500	0.640 (0.332-1.230)	0.180	0.517 (0.231-1.160)	0.109
White blood cell count (10^^^9/L)	1.013 (0.829-1.237)	0.903		
Lymphocyte count (10^^^9/L)	0.979 (0.944-1.016)	0.266	0.972 (0.918-1.031)	0.350
Platelet count (10^^^9/L)	1.002 (0.998-1.006)	0.378		
Alanine aminotransferase (U/L)	0.990 (0.969-1.010)	0.333		
Aspartate aminotransferase (U/L)	0.990 (0.967-1.014)	0.403		
B cells (% of lymphocytes)	0.989 (0.884-1.105)	0.841		
RBD-specific B cells (%)	0.984 (0.919-1.053)	0.638		
RBD-specific MBCs (%)	0.985 (0.959-1.013)	0.294	0.161 (0.023-1.138)	0.067
RBD^+^ rMBCs (%)	0.973 (0.929-1.020)	0.264	0.163 (0.0001-241.290)	0.626
RBD^+^ actMBCs (%)	0.982 (0.932-1.035)	0.491	0.184 (0.0001-274.513)	0.650
RBD^+^ atyMBCs (%)	1.002 (0.953-1.053)	0.936	0.027 (0.00001-53.250)	0.351
RBD^+^ intMBCs (%)	1.017 (0.986-1.049)	0.285	0.028 (0.00001-55.202)	0.355

CI, confidential interval; OR, odds ratio.

## Discussion

In this prospective study, we evaluated the safety, antibody responses, and RBD-specific MBC responses of inactivated SARS-CoV-2 vaccines in PLWH and healthy controls. Our results showed that inactivated vaccines were safe and well-tolerated in PLWH. The antibody and MBC responses waned in PLWH, especially in PLWH with CD4 counts <200 cells/µL. Therefore, PLWH should be vaccinated ahead of the healthy population.

PLWH with SARS-CoV-2 have poor clinical outcomes, especially those who are immunosuppressed or do not receive ART [18,19]. However, the registration trials of the inactivated SARS-CoV-2 vaccines on the safety and humoral immune response in HIV-infected populations are limited. Hence, we first assessed the safety of inactivated vaccines in PLWH. The overall incidence of adverse events within 7 days in PLWH was 12.9%, which was similar to healthy controls (13.3%), but it was lower than phase 1/2 trials of BBIBP-CorV in China [16] (23–29%) and phase 3 trials of Corona Vac in Turkey [17] (18.9%). Notably, the proportion of adverse reactions in PLWH who received the inactivated vaccines is significantly lower than that in PLWH who received mRNA vaccines (60%) [10]. This discrepancy may be attributed to the different types of vaccines. No serious adverse events occurred after inoculation of the inactivated vaccines in PLWH, suggesting that inactivated vaccines are safe and reliable.

Our results showed that PLWH had lower anti-RBD-IgG and anti-spike-IgG titers than healthy controls 21–105 days after vaccination, which is consistent with previous studies showing that PLWH had lower immune responses to the vaccine than healthy individuals [20–22]. Previous studies also showed that 3–4 weeks after the first dose of vaccination, the production of anti-RBD antibodies in PLWH was lower than in HIV-negative controls. However, after the second dose of vaccination, the antibody levels did not differ significantly between the two groups [11,23,24]. This difference may be due to the type of vaccine, the population included, and the duration of ART. Thus, more data will be needed to clarify this in the future.

The level of immunosuppression is usually reflected by the CD4 cell counts [25]. Usually, HIV infection will cause the gradual loss of CD4^+^ T cells and a series of immune abnormalities [26]. However, after highly active ART, the CD4 counts of some patients can gradually increase [27]. In this study, PLWH with CD4 counts <200 cells/µL had significantly lower anti-RBD-IgG levels, similar to previous studies [10,11]. This suggests that effective ART for PLWH is needed to strengthen immunity against SARS-CoV-2. In addition, both cross-sectional and longitudinal analyses showed that the antibody titers in PLWH declined over time, which is similar to the results of previous longitudinal studies using mRNA vaccines [28–30]. Of note, anti-RBD-IgG and anti-spike-IgG titers in PLWH were lower than the titers in healthy controls at every time point in the cross-sectional analysis. Considering the occurrence of breakthrough infections with SARS-CoV-2 was correlated with low neutralizing antibody titers [31], hence, more concern should be taken on this special population.

The MBCs produced during primary infection are quickly reactivated after a secondary infection, thus, preventing severe disease or death [14,15]. Hence, we focused on the response of MBCs to inactivated vaccines. In the cross-sectional analysis, the frequency of RBD-specific MBCs in PLWH was lower than that in healthy controls, but it was relatively stable over time, which was corroborative in the longitudinal analysis. This suggests that the durable humoral immunity may be dysfunctional. In addition, the frequency of actMBCs was reduced in PLWH. ActMBCs are cells that recently left germinal centres and are already primed to become antibody-secreting plasma cells [32]. This suggests that the immune reactivation after inactivated SARS-CoV-2 vaccination may be impaired in PLWH.

There are some limitations in this study. First, few subjects participated in the follow-up until month 6 after full vaccination because the sporadic localized outbreaks of COVID-19 partially impeded travel. Second, the early stages of B and T cell responses were not evaluated, as we were mainly focusing on the significance of durable humoral immune responses. Therefore, further studies about the early stages of B and T cell responses in PLWH are needed. Third, the best correlation of antibody titers and vaccine efficacy in PLWH is currently unknown, and more large-scale population studies are needed. Nonetheless, we believe our results are particularly important and meaningful to clinicians.

In conclusion, the inactivated SARS-CoV-2 vaccines are safe and well-tolerated in people living with HIV, with no serious adverse events reported. However, the antibody response and RBD-specific MBC response were weak, especially in PLWH with a low CD4 count. Therefore, SARS-CoV-2 vaccines and booster doses should be prioritized for this special population.

## Supplementary Material

Supplemental Material
