# Supplementary material for: Safety and immunogenicity of inactivated SARS-CoV-2 vaccines in people living with HIV
Source: Emerg Microbes Infect. 2022 Apr 18;11(1):1126–34. doi: 10.1080/22221751.2022.2059401 (PMC9037169; doi:10.1080/22221751.2022.2059401)
Supplement: Supplemental Material [file TEMI_A_2059401_SM7958.docx]

**Safety and** **immunogenicity of inactivated SARS-CoV-2 vaccines in people living with HIV**

Ling Ao ^a#^, TingLu ^a#^, YuCao^b#^, Zhiwei Chen ^a#^, Yuting Wang^a^, Zisheng Li^b^, Xingqian Ren^b^, PanXu^a^, Mingli Peng^a^, Min Chen^a^, Gaoli Zhang^a^, Dejuan Xiang^a^, Dachuan Cai^a^, Peng Hu^a^, Xiaofeng Shi^a*^, Dazhi Zhang ^a*^, Hong Ren^a*^

Table of contents

Supplementary methods....................................................................................3

Supplementary Figure1.....................................................................................4

Supplementary Figure 2....................................................................................5

Supplementary Figure 3....................................................................................6

Supplementary Figure 4....................................................................................7

Supplementary Figure 5....................................................................................8

**Supplementary methods**

## Evaluation of SARS-CoV-2 RBD binding antibody

An indirect ELISA was performed to detect anti-receptor binding domain (RBD)-IgG antibodies according to the manufacturer's instructions (Sino Biological, Beijing, China). Briefly, plate wells were pre-coated with 0.5 ug/mL recombinant RBD protein (100 μL per well) and incubated overnight at 4 °C. After completely discarding the intraplate solution, 300 μL 6% bovine serum albumin (BSA) solution was added to each well and incubated at room temperature for 1 h. After thoroughly cleaning the well, a continuously diluted sample or control (100 µL) was added, mixed thoroughly, and incubated at room temperature for 2 h. After washing the plate 3 times, the goat anti-human IgG secondary antibody (100 μL/well) was coupled with diluted horseradish peroxidase (HRP), mixed, and incubated at room temperature for 1 h. After washing and adding the substrate (TMB) and stop solutions, absorbance (OD value) was read at 450 nm. Starting at 1:50, the serum sample was diluted with a double series of diluents. In each plate, continuously diluted positive antibody controls (anti-RBD antibodies) and negative controls (sera from individuals with no history of SARS-CoV-2 infection and vaccination) were detected simultaneously. ELISA measurements were made in duplicate. When the OD value of the 1:50 dilutions ≥2.1 times the average absorbance of the negative control, the serum was positive for IgG binding antibodies. The antibody level was expressed as the highest serum dilution showing positive results. The kit reported that anti-RBD-IgG tests have 100% sensitivity and 98% specificity for the diagnosis of COVID-19.

## Evaluation of anti-spike-IgG

An ELISA was performed to detect anti-spike-IgG antibodies according to the manufacturer's protocol (Sino Biological, Beijing, China). Briefly, plate wells were pre-coated with 0.5 ug/mL recombinant spike (S1+S2) protein (100 μL per well) and incubated at 4 ℃ overnight. After thoroughly discarding solutions in the plate, 300 μL of 6% BSA solution was added to each well and incubated for 1 h at room temperature. After washing wells thoroughly, samples (1:50 dilute) or controls (100 μL) were added, mixed well, and incubated for 2 h at room temperature. Following three times of washing plates, diluted HRP-conjugated goat anti-human IgG secondary antibody was added (100 μL per well), mixed well, and incubated for 1 h at room temperature. After washing and adding substrate (TMB) and stop solutions, absorbance was read at 450 nm. All measurements were performed in duplicate. A positive result for the anti-spike-IgG was defined as >1.495 AU/mL. The kit reported that anti-spike-IgG tests have 100% sensitivity and 98% specificity.

**
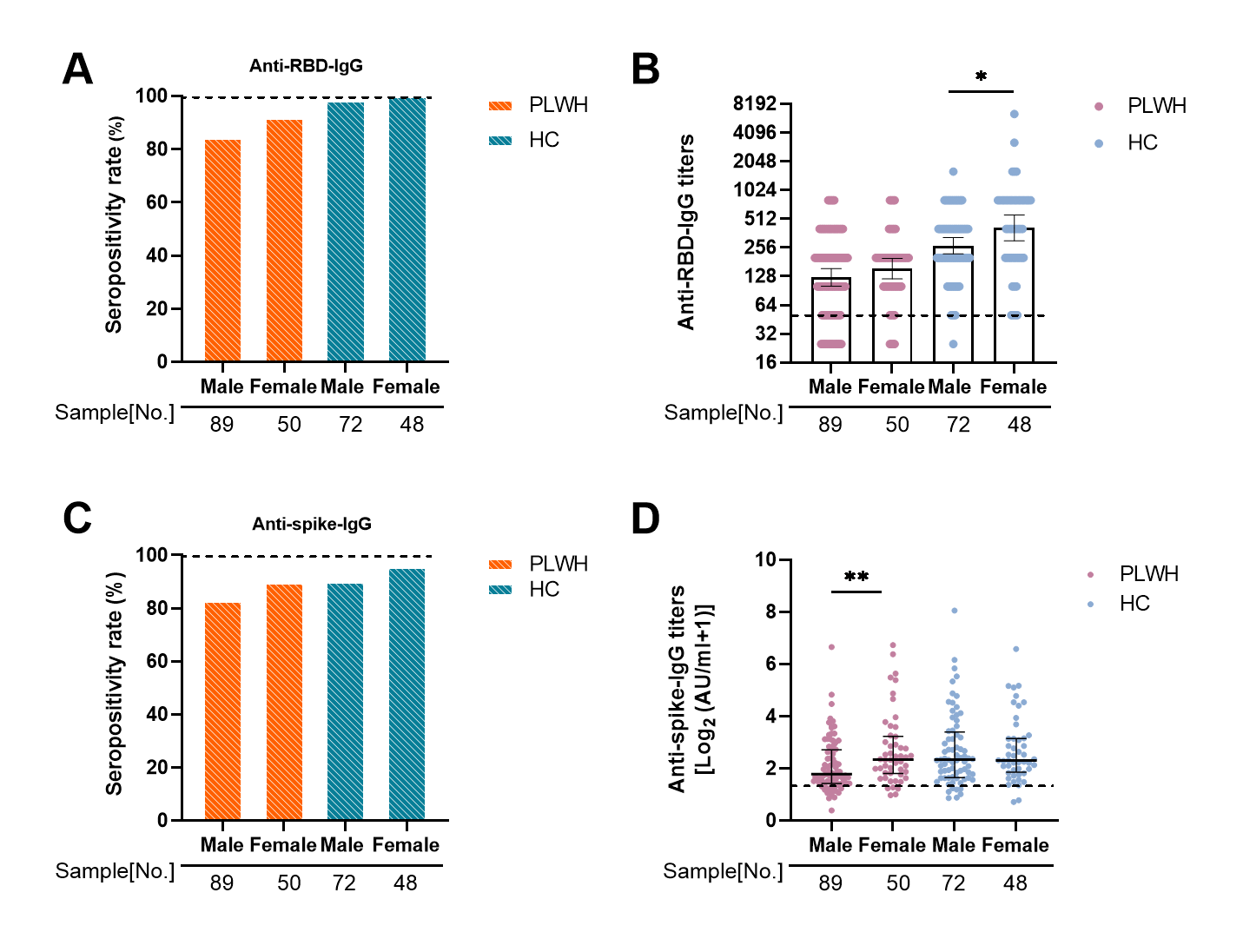
**

**Supplementary Figure 1. Gender-stratified antibody responses to inactivated vaccines in people living with HIV (PLWH).** The seropositivity rate (**A**) and titers (**B**) of anti-receptor binding domain (RBD)-IgG in PLWH and healthy controls. The seropositivity rate (**C**) and titers (**D**) of anti-spike-IgG in PLWH and healthy controls. The horizontal dotted lines represent the limit of detection.

**
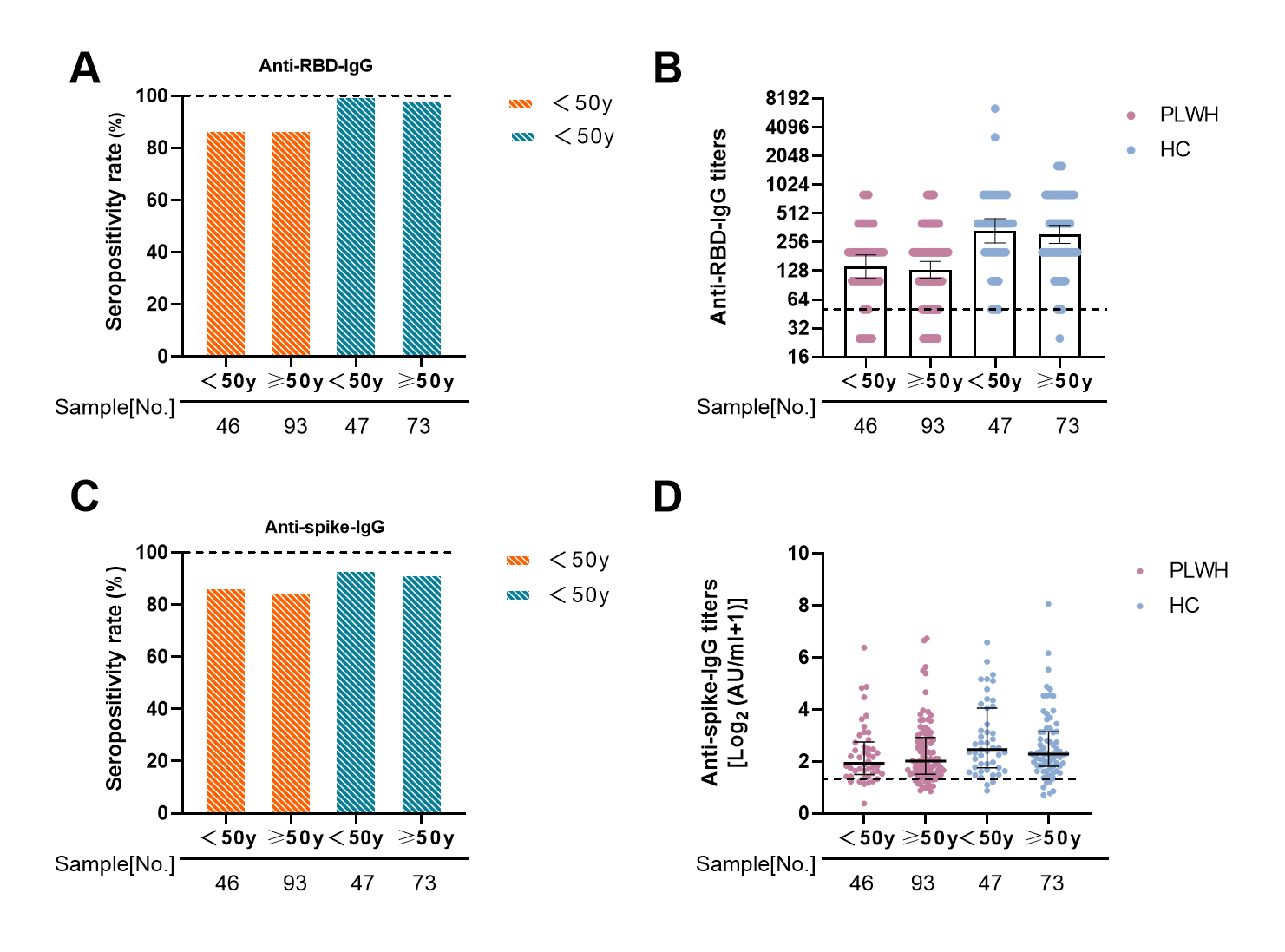
**

**Supplementary Figure 2. Age-stratified antibody responses to inactivated vaccines in people living with HIV (PLWH).** The seropositivity rate (**A**) and titers (**B**) of anti-receptor binding domain (RBD)-IgG in PLWH and healthy controls. The seropositivity rate (**C)** and titers (**D**) of anti-spike-IgG in PLWH and healthy controls. The horizontal dotted lines represent the limit of detection.

**
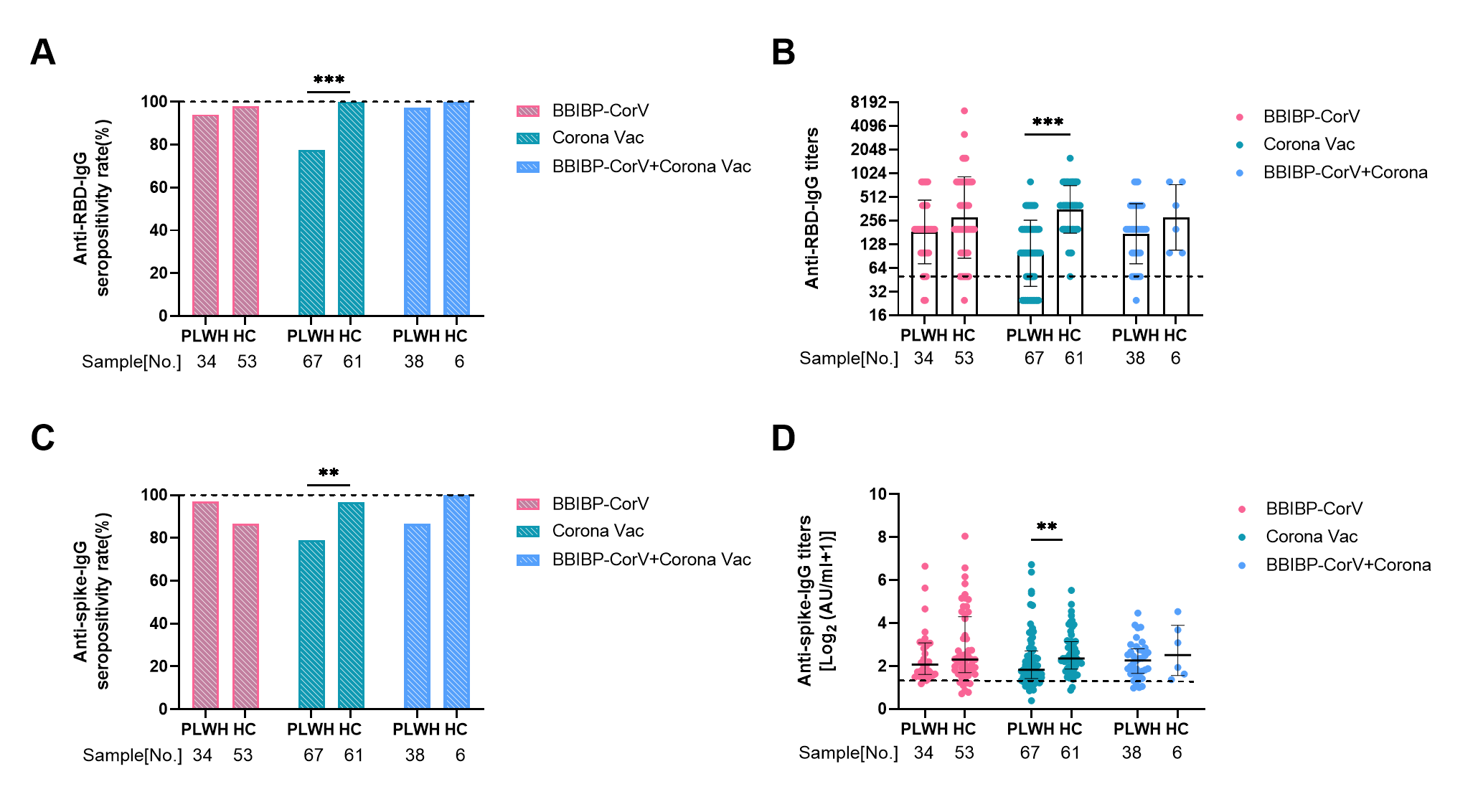
**

**Supplementary Figure 3.Antibody responses to different types of inactivated vaccines in people living with HIV (PLWH).** The seropositivity rate (**A**) and titers (**B**) of anti-receptor binding domain (RBD)-IgG in PLWH and healthy controls vaccinated with BBIBP-CorV, Corona Vac, or BBIBP-CorV+Corona Vac vaccines, respectively. The seropositivity rate (**C**) and titers (**D**) of anti-spike-IgG in PLWH and healthy controls vaccinated with BBIBP-CorV, Corona Vac, or BBIBP-CorV+Corona Vac vaccines, respectively. The horizontal dotted lines represent the limit of detection.

**
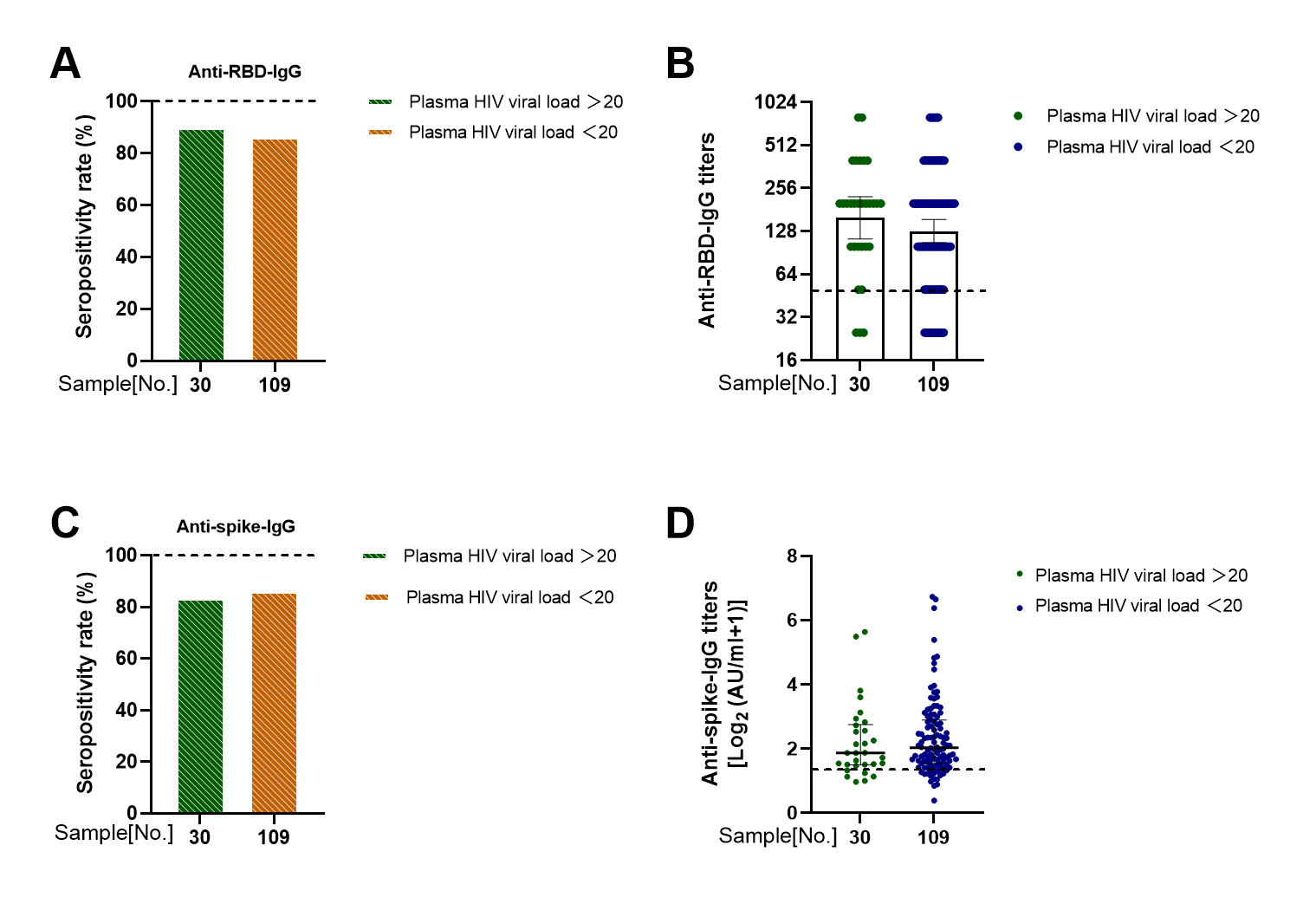
**

**Supplementary Figure 4. Antibody responses to inactivated vaccines in people living with HIV (PLWH) with different viral loads.** The seropositivity rate (**A**) and anti-receptor binding domain (RBD)-IgG titers (**B**) in PLWH with different plasma HIV viral loads. The seropositivity rate (**C**) and anti-spike-IgG titers (**D**) in PLWH with different plasma HIV viral loads. The horizontal dotted lines represent the limit of detection.


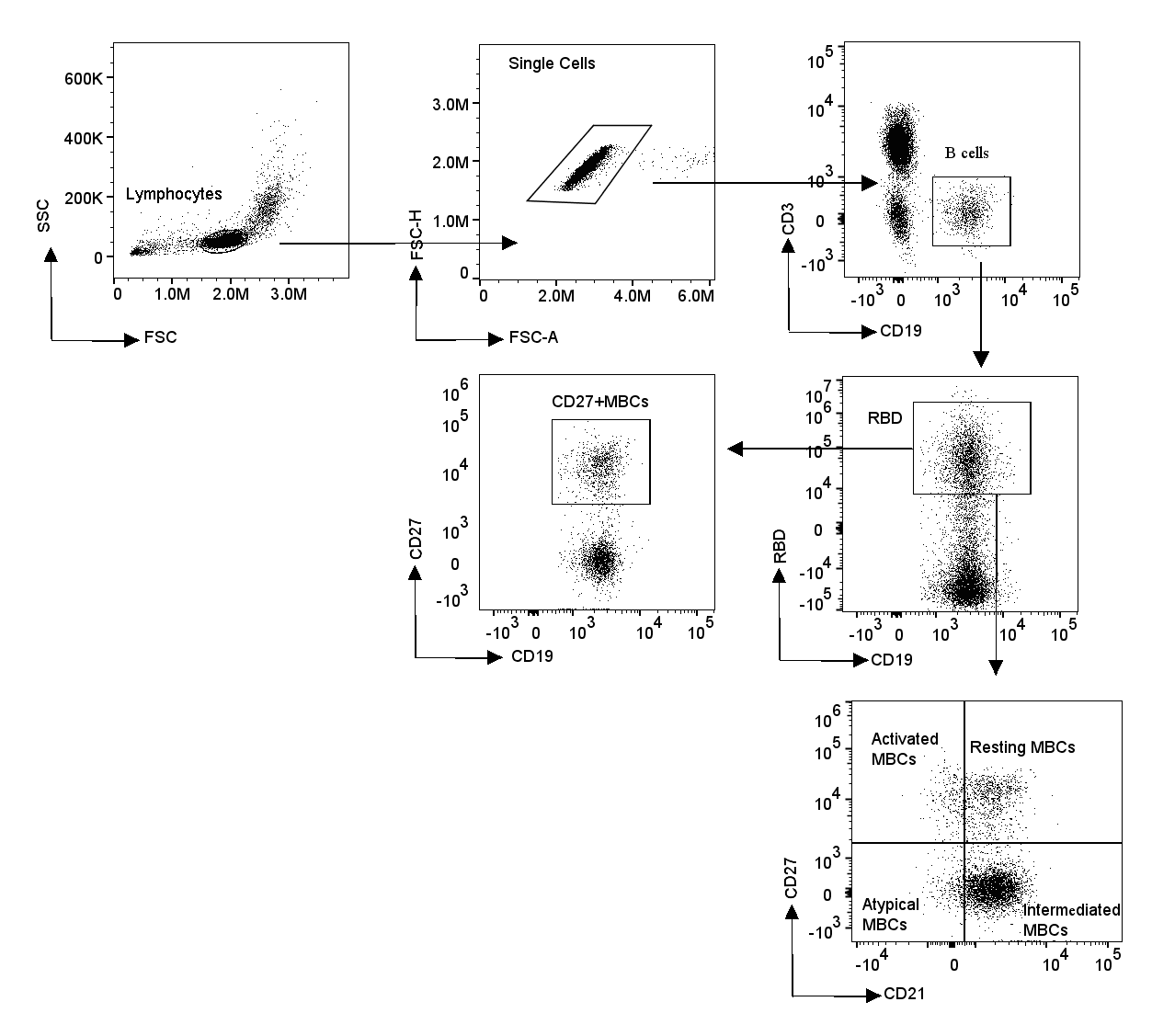
**Supplementary Figure 5. Full gating strategy of flow cytometry for target cell population.**
